# Cryptococcus neoformans infection in organ transplant recipients: variables influencing clinical characteristics and outcome.

**DOI:** 10.3201/eid0703.010302

**Published:** 2001

**Authors:** S Husain, M M Wagener, N Singh

**Affiliations:** Veterans Affairs Medical Center and University of Pittsburgh, Thomas E. Starzl Transplantation Institute, Pittsburgh, Pennsylvania 15240, USA.

## Abstract

Unique clinical characteristics and other variables influencing the outcome of Cryptococcus neoformans infection in organ transplant recipients have not been well defined. From a review of published reports, we found that C. neoformans infection was documented in 2.8% of organ transplant recipients (overall death rate 42%). The type of primary immunosuppressive agent used in transplantation influenced the predominant clinical manifestation of cryptococcosis. Patients receiving tacrolimus were significantly less likely to have central nervous system involvement (78% versus 11%, p =0.001) and more likely to have skin, soft-tissue, and osteoarticular involvement (66% versus 21%, p = 0.006) than patients receiving nontacrolimus- based immunosuppression. Renal failure at admission was the only independently significant predictor of death in these patients (odds ratio 16.4, 95% CI 1.9-143, p = 0.004). Hypotheses based on these data may elucidate the pathogenesis and may ultimately guide the management of C. neoformans infection in organ transplant recipients.


### *Cryptococcus neoformans* Infection in Organ Transplant Recipients: Variables Influencing Clinical Characteristics and Outcome

**Shahid Husain, Marilyn M. Wagener, and Nina Singh**
Veterans Affairs Medical Center and University of Pittsburgh,
Thomas E. Starzl Transplantation Institute, Pittsburgh, Pennsylvania, USA

Unique clinical characteristics and other variables influencing the outcome of *Cryptococcus neoformans* infection in organ transplant recipients have not been well defined. From a review of published reports, we found that *C. neoformans* infection was documented in 2.8% of organ transplant recipients (overall death rate 42%). The type of primary immunosuppressive agent used in transplantation influenced the predominant clinical manifestation of cryptococcosis. Patients receiving tacrolimus were significantly less likely to have central nervous system involvement (78% versus 11%, p =0.001) and more likely to have skin, soft-tissue, and osteoarticular involvement (66% versus 21%, p = 0.006) than patients receiving nontacrolimus-based immunosuppression. Renal failure at admission was the only independently significant predictor of death in these patients (odds ratio 16.4, 95% CI 1.9–143, p = 0.004). Hypotheses based on these data may elucidate the pathogenesis and may ultimately guide the management of *C. neoformans* infection in organ transplant recipients.

Invasive fungal infections have been reported in 5% to 59% of organ transplant recipients (1-4). Infections due to *Cryptococcus neoformans*, while less common than those due to *Candida* and mycelial fungi, are also an important posttransplant complication. The incidence of invasive candidiasis has declined in subsets of organ transplant recipients (e.g., liver transplant patients) as a result of fluconazole use and technologic advances in surgery (5). However, the risk factors and pathogenesis of *C. neoformans* infection in the transplant setting are poorly understood, and fluconazole prophylaxis is generally not used in the late posttransplant period when cryptococcal infections usually occur. Thus, the incidence and impact of cryptococcal infection in organ transplant recipients are unlikely to diminish in the foreseeable future. Indeed, as incidence of *C. neoformans* infection in HIV-infected patients has declined, organ transplant recipients have become the group of immunocompromised patients at highest risk for cryptococcosis. The overall death rate in transplant recipients with cryptococcal infection has been 20% to 100% (6-9). While the predictors of outcome in patients with *C. neoformans* have been well documented in nontransplant settings (10-12), predictors in transplant recipients are largely unknown. The unique neurotropism and predilection of *C. neoformans* to cause central nervous system (CNS) infections are well recognized; CNS has been the most common site for cryptococcal infections. However, 67% of our liver transplant recipients with cryptococcosis who received tacrolimus as primary immunosuppression had cutaneous or osteoarticular lesions; 17% had meningitis (8). Small sample size and lack of comparison with patients on other immunosuppressive regimens, however, precluded meaningful interpretation of these data.

Given the limited number of transplant recipients with *C. neoformans* infection at individual institutions, accumulating a sufficiently large sample was difficult, so we turned to reports and analyses of cases for valuable data. This review summarizes unique epidemiologic and clinical characteristics of *C. neoformans* in transplant recipients, as well as variables influencing the outcome of cryptococcal infections after transplantation.

Address for correspondence: Nina Singh, VA Medical Center, Infectious Disease Section, University Drive C, Pittsburgh, PA 15240, USA; fax: 412-688-6950; e-mail: nis5+@pitt.edu

### Methods

Cases of *C. neoformans* infection in transplant recipients were identified with a MEDLINE search through 1998 by cross-referencing the keywords "*Cryptococcus neoformans*" and "transplantation" or "transplant." Reference lists of original articles and textbooks were reviewed for additional cases. A patient was considered infected if *C. neoformans* was cultured from a clinical specimen in the presence of signs or symptoms of cryptococcus infection. The onset of infection after transplantation was determined on the basis of detailed case studies; summarized data providing only a mean or range for the group of transplant recipients were excluded. Cryptococcal infection was considered early-onset if it occurred within 12 months and late-onset if it occurred >12 months after transplantation. Predictors or risk factors for death were assessed only in detailed cases for which the variables to be analyzed were explicitly stated.

### Statistical Analysis

Patient demographic data were entered into the database PROPHET Statistics Version 5.0 (BBN Systems and

Technologies, Cambridge, MA). The $\chi^3$ or the Fisher exact test was used to compare categorical variables. Continuous variables (e.g., time of onset) were compared by using the Student *t* test or the Mann-Whitney U test. Multiple comparisons were done by analysis of variance and the Kruskal-Wallis test. A multiple regression model was used to examine the risk factors for death.

### Results

A total of 178 cases of *C. neoformans* infection in organ transplant recipients were identified (1,6-9,13-56). Of these, 96 cases were individually detailed, and 82 were summarized in reports containing 2 to 22 cases. Of 178 cases, 145, 20, and 10 were in renal, liver, and heart transplant recipients, respectively. Three cases were reported in lung transplant recipients, and none were described in bowel or pancreas transplant recipients. Patients were 12 to 67 years of age (median 44 years); 78% were male. The mean incidence of *C. neoformans* infection was 2.8 per 100 transplants (0.3 to 5.3 per 100). The overall incidence was 2.4% in liver, 2.0% in lung, 3.0% in heart, and 2.8% in renal transplant recipients.

Of 127 transplant recipients who could be evaluated, 100 (79%) had azathioprine as the primary immunosuppressive agent, 9 (7%) had tacrolimus, 11 (9%) had cyclosporine, and 7 (6%) had cyclosporine and azathioprine. Of these 127 patients, 78 were also receiving prednisone in various dosages, 5 were not receiving prednisone, and data on prednisone use were unavailable for 44 patients. The incidence of cryptococcosis was 4.5 per 100 transplants in patients who received tacrolimus, 2.4 per 100 transplants in patients who received cyclosporine, and 3.4 per 100 transplants in patients who received azathioprine. These rates did not differ significantly. Rejection episodes preceding cryptococcal infection were documented in 17 (25%) of 67 patients; rejection had occurred a median of 7 months (from 5 days to 49 months) before onset of infection. Eleven (18%) of 62 patients had received augmented immunosuppression (predominantly corticosteroids) within 6 months of onset of cryptococcosis; two patients had received antilymphocyte preparations or OKT3 monoclonal antibodies for the treatment of allograft rejection.

### Time to Onset

Cryptococcosis occurred a median of 1.6 years (from 2 days to 12 years) after transplantation. Overall, 14 (15%) of 94 cases occurred within 3 months, 10 (11%) of 94 in 3 to 6 months, 15 (16%) of 94 in 6 to 12 months, and 55 (59%) of 94 >12 months after transplantation.

The time to onset varied significantly for different types of organ transplant recipients. The median time to onset after transplantation was 35 months for kidney, 25 months for heart, 8.8 months for liver, and 3 months for lung transplant recipients (p = 0.001). Overall, cryptococcosis developed in 100% of the lung, 75% of the liver, 33% of the heart, and 30% of the kidney transplant recipients within 12 months of transplantation (p = 0.002) (Table 1).

*C. neoformans* infection tended to occur later in patients who received azathioprine than in patients who received tacrolimus or cyclosporine (p = 0.16). The median time to onset was 11.4 months after transplantation in patients who received cyclosporine, 9.2 months in patients who received tacrolimus, and 27 months in patients who received only azathioprine-based immunosuppression (p = 0.16). Patients from the northeastern United States were more likely to have early-onset cryptococcosis (i.e., infection within 12 months of transplantation) than other patients (67% versus 31%, p = 0.004). Age, cytomegalovirus infection, or prior rejection episodes did not correlate with early- versus late-onset cryptococcal infection (Table 1).

Table 1. Variables associated with early and late-onset *Cryptococcus neoformans* infection in organ transplant recipients

| Variable (no. of patients for whom data available) | Early onset (within 12 months) (%) | Late onset (>12 months) (%) | p value |
|---|---|---|---|
| Mean age in yrs | 42.2 | 44.3 | NS[a] |
| Type of transplant | | | 0.001 |
| Liver (20) | 75 | 25 | |
| Kidney (54) | 28 | 72 | |
| Heart (9) | 33 | 67 | |
| Lung (2) | 100 | 0 | |
| Cytomegalovirus (CMV) infection (2) | 50 | 50 | NS |
| No CMV infection (6) | 67 | 33 | |
| Prior rejection (16) | 50 | 50 | NS |
| No prior rejection (45) | 36 | 64 | |
| U.S. region | | | 0.004[b] |
| Northeast (24) | 67 | 33 | |
| West (19) | 32 | 68 | |
| Midwest (9) | 22 | 78 | |
| South (20) | 40 | 60 | |
| Other countries | | | |
| Europe (7) | 28 | 71 | |
| Asia (3) | 33 | 67 | |
| Site involved | | | |
| Lung (26) | 42 | 58 | NS |
| Central nervous system (57) | 40 | 60 | |
| Skin/osteoarticular (23) | 30 | 70 | |
| Death rate (84) | 34 | 41 | |

[a]NS = not significant, p >0.05.
[b]Northeastern United States versus all other regions.

### Clinical Manifestations

Of 159 patients, 87 (55%) had *C. neoformans* infection at the CNS site only; 20 (13%) had skin, soft tissue, or osteoarticular infection only; and 10 (6%) had pulmonary infection only. One patient each had prostate gland infection, myositis, chorioretinitis, and isolated renal allograft involvement due to *C. neoformans* (14,15,19,32). In 38 (24%) of the 159 patients, more than one site of infection was documented: CNS in 115 (72%) of 159; pulmonary in 39 (25%) of 159; and skin, soft tissue, or osteoarticular involvement in 34 (21%) of 159 patients.

Patients receiving tacrolimus were significantly less likely to have CNS involvement than patients receiving nontacrolimus-based immunosuppression (78% versus 11%, p = 0.013). Skin, soft-tissue, or osteoarticular involvement was significantly more likely to occur with a tacrolimus- (66%) than with a nontacrolimus-based immunosuppressive regimen (21%, p = 0.006). When patients who received tacrolimus were compared with those who received cyclosporine, CNS involvement (1 [11%] of 9 versus 12 [67%] of 18, p = 0.01) was significantly lower, and skin, soft-tissue, or osteoarticular involvement was significantly higher with tacrolimus than with cyclosporine immunosuppressive therapy (6 [67%] of 9 versus 4 [22%] of 18, p = 0.04).

Positive blood cultures for *C. neoformans* were documented in 15 (38%) of 39 transplant recipients for whom blood cultures were performed. However, 32 (91%) of 35 patients for whom serum cryptococcal antigen was performed had a positive serum cryptococcal antigen of 1:2 to 1:8192 (median 1:256). Leukocytosis was largely absent, the mean peripheral leukocyte count of the patients in this review was 6,560/$mm^3$ (range 2,000 to 12,000/$mm^3$). Sixty-eight (74%) of 91 patients were febrile.

### CNS Infection

Of 125 patients with CNS involvement (6,7,9,13,14,16,20,22,23,26,30,31,37-39,42,43,45-47,49-51,53,57), 122 (98%) had meningitis. Space-occupying lesions (contrast enhancing mass lesions) due to *C. neoformans* were present in three patients (7,23). Thirty-nine (62%) of 63 patients with CNS cryptococcosis had headache, 30 (48%) of 62 had confusion or lethargy, and 2 (1%) of 25 had coma on admission. Serum cryptococcal antigen was positive in 18 (86%) of 21 patients with CNS infection (median titer 1:256; range 1:4 to 1:4096). However, 100% of 37 patients had a positive CSF cryptococcal antigen (median titer 1:256; range 1:4 to 1:32,768). CSF cultures yielded *C. neoformans* in 76 (93%) of 82 patients, and India ink preparation was positive in 36 (77%) of 47 patients with CNS infection (Table 2).

Table 2. Cerebrospinal fluid (CSF) characteristics in organ transplant recipients with central nervous system *Cryptococcus neoformans* infection

| Variable (no. of patients whom data available) | Value[a] |
|---|---|
| Opening pressure, mm $H_20$ (17) | 330 (140-700) |
| Leukocytes, $mm^3$ (27) | 33 (0-485) |
| Protein, mg/dL (27) | 74 (16-715) |
| Glucose, mg/dL (27) | 36 (4 – 113) |
| No. with positive India ink | 80% (38/47) |
| No. with positive CSF cryptococcal antigen | 100% (27/27) |
| Titer, median (range) | 1:512 (1:4-1:32,768) |
| No. with positive CSF culture | 93% (76/82) |
| No. with positive serum cryptococcal antigen | 88% (14/16) |
| Titer, median (range) | 1:128 (1:4-1:4096) |

[a]Median and range unless otherwise stated.
[b]Numbers of patients for whom data were available.

### Pulmonary Infection

Unilateral, nodular, or cavitary infiltrates were the most frequent radiographic signs of pulmonary cryptococcosis (1,7,9,13,23,26,29,37-40,46,49,50,54-56). Pleural effusions were documented in 4 of 42 patients. Serum cryptococcal antigen was detectable in 100% of 12 patients with pulmonary lesions (titers of 1:4 to 1:8192).

### Skin, Soft Tissue, or Osteoarticular Infection

Seventy-two percent of patients with cutaneous cryptococcosis (6,9,13,16,17,21,22,25,27-29,35-37,40,44,46,49,54-56,58) had cellulitis; *C. neoformans* was cultured from an aspirate or biopsy in all these cases. Other signs included papular or nodular lesions. Septic arthritis and osteomyelitis were documented in five cases. Nineteen (90%) of 21 patients with skin or osteoarticular cryptococcal infections had positive serum cryptococcal antigen.

### Death Rate

The overall death rate among organ transplant recipients with cryptococcal infection was 72 (42%) of 172. The death rate was 8 (40%) of 20 for liver, 57 (41%) of 139 for kidney, 6 (60%) of 10 for heart, and 1 (33%) of 3 for lung transplant recipients. Death rates did not differ between patients on tacrolimus and patients on other primary immunosuppressive regimens (33% versus 38%, $p > 0.05$). CNS infection ($p = 0.04$), renal failure (defined as serum creatinine >1.5 mg/dL on admission, $p = 0.005$), and abnormal mental status ($p = 0.03$) were significant predictors of death in univariate analysis (Table 3). In logistic regression analysis (with the above variables in the model), only renal failure on admission was predictive of death (odds ratio 16.4; 95% CI 1.9 to 143; $p = 0.004$). The death rate was 25 (48%) of 52 in patients receiving amphotericin B deoxycholate, 29 (38%) of 77 in patients receiving amphotericin B plus 5 flucytosine, and 3 (21%) of 14 in patients receiving fluconazole ($p = 0.16$). Fluconazole, however, was less likely to be used in patients with CNS infection; 5% of patients with CNS compared with 23% of those with extraneural infection had received fluconazole ($p = 0.01$).

Forty-nine (49%) of 101 patients with CNS cryptococcal infection died. Of 79 patients with CNS infection who received an antifungal agent, 22 had received amphotericin B alone, 52 had received amphotericin B plus 5-flucytosine, and 5 had received fluconazole. Death rates did not differ between patients with CNS infection who received amphotericin B alone (59%) and patients with CNS infection who received amphotericin B plus flucytosine (44%). Abnormal mental

Table 3. Variables associated with death in organ transplant recipients with *Cryptococcus neoformans* infection

| Variable (no of patients for whom data available) | Death (%) | Survival (%) | p value |
|---|---|---|---|
| Mean age in yrs | 43.6 | 43.4 | NS[a] |
| Prior rejection (17) | 35 | 65 | NS |
| No rejection (50) | 28 | 72 | |
| Rejection within 6 months of onset of cryptococcosis (3) | 33 | 67 | NS |
| Increased immunosuppression (11) | 46 | 54 | NS |
| Fever (39) | 31 | 69 | NS |
| No fever (21) | 33 | 67 | |
| Renal failure (37) | 43 | 57 | 0.005 |
| No renal failure (18) | 6 | 94 | |
| Mental status | | | |
| Abnormal (22) | 54 | 45 | 0.03 |
| Normal (53) | 28 | 72 | |
| Treatment | | | NS |
| AmB[b] (52) | 48 | 52 | (0.16) |
| AmB + 5 FC[c] (77) | 38 | 62 | |
| Fluconazole (14) | 21 | 79 | |
| Site involved | | | 0.04[d] |
| Central nervous system (101) | 49 | 51 | |
| Pulmonary (32) | 22 | 78 | |
| Skin/osteoarticular (28) | 21 | 79 | |
| Type of transplant | | | NS |
| Liver (20) | 40 | 60 | |
| Kidney (139) | 41 | 59 | |
| Heart (10) | 60 | 40 | |
| Lung (3) | 33 | 67 | |

[a]NS = not significant, $p > 0.05$.
[b]AmB = amphotericin B deoxycholate.
[c]FC = flucytosine.
[d]p value represents the difference for CNS versus other sites.

status and absence of headache (p = 0.07) correlated with poor outcome in patients with CNS cryptococcal infection (Table 4). Presence of fever, CSF pleocytosis, positive blood cultures, and CSF cryptococcal antigen titer did not correlate with outcome (Table 4).

Table 4. Variables associated with death in patients with central nervous system *Cryptococcus neoformans* infection

| Variable (no. of patients for whom data available) | Death (%) | Survival (%) | p value |
|---|---|---|---|
| Mean age in yrs | 40.6 | 42.4 | NS[a] |
| Fever (29) | 34 (10/29) | 66 (10/29) | NS |
| No fever (7) | 43 (3/7) | 57 (4/7) | |
| Headache (20) | 25 (5/20) | 75 (15/20) | NS |
| No headache (21) | 52 (11/21) | 48 (10/21) | (0.09) |
| Abnormal mental status (20) | 55 (11/20) | 45 (9/20) | NS |
| Normal mental status (26) | 31 (8/26) | 69 (18/26) | |
| White blood cell >20/mm$^3$ (20) | 40 (8/20) | 60 (12/20) | NS |
| White blood cell <20/mm$^3$ (13) | 62 (8/13) | 38 (5/13) | |
| Cryptococcal antigen titer ≥1,024 (10) | 20 (2/10) | 80 (8/10) | NS |
| Cryptococcal antigen titer <1,024 (17) | 35 (6/17) | 65 (11/17) | |
| Positive blood culture (8) | 13 (1/8) | 87 (7/8) | NS |
| Negative blood culture (16) | 50 (8/16) | 50 (8/16) | |
| Renal failure (22) | 54 (12/22) | 46 (10/22) | 0.011 |
| No renal failure (12) | 8 (1/12) | 92 (11/12) | |
| Therapy | | | NS |
| AmB[b] alone (55) | 47 (26/55) | 53 (29/55) | |
| AmB + 5 FC[c] (32) | 50 (16/32) | 50 (16/32) | |
| Fluconazole (5) | 40 (2/5) | 60 (3/5) | |

[a]NS = not significant, p >0.05.
[b]AmB = Amphotericin B deoxycholate.
[c]FC = flucytosine.

### Discussion

*C. neoformans* infection was documented in 2.8% of the organ transplant recipients, with an overall death rate of 42%. A number of findings in our study have previously not been fully appreciated in the context of cryptococcal infections after transplantation. For example, the type of primary immunosuppression after organ transplantation may influence the predominant clinical manifestation. Patients receiving tacrolimus were less likely to have CNS involvement and more likely to have skin, soft tissue, or osteoarticular involvement due to *C. neoformans* than patients who received nontacrolimus-based immunosuppression. Furthermore, both tacrolimus and cyclosporine were less likely to be associated with CNS involvement and more likely to be associated with cutaneous infection than azathioprine.

A number of biologic plausibilities exist for this observation. Tacrolimus is a natural macrolide antifungal product (59,60). Although its immunosuppressive effect outweighs its antifungal action in vivo, tacrolimus is toxic to *C. neoformans* in vitro by inhibition of calcineurin (59-61). Furthermore, tacrolimus suppresses the growth of *C. neoformans* at 37°C but not at 24°C, which suggests that the target of tacrolimus, calcineurin, is required at higher body temperatures (59,61). Thus, temperature-dependent inhibition of cryptococci by tacrolimus may prevent CNS infection but allow growth of fungus at cooler body sites, e.g., skin, soft tissue, and bone. Cyclosporine also possesses in vitro antifungal activity by inhibition of calcineurin (60,61). However, cyclosporine does not effectively penetrate the CNS, while tacrolimus crosses the blood-brain barrier (61,62). Thus, the relative rarity of meningitis compared with extraneural manifestations of cryptococcosis in patients receiving tacrolimus may merely be due to high cerebrospinal fluid levels of tacrolimus.

Strains of *C. neoformans* known to be selectively dermatotropic and rhinotropic have been demostrated in animal models (63,64). In addition, *C. neoformans* serotype D is more likely to be associated with cutaneous lesions (65). However, the precise reason for dermatotropism or the propensity of these strains to occur in transplant recipients receiving calcineurin-inhibiting agents (e.g., cyclosporine and tacrolimus) has not been elucidated.

The immunosuppressive agents (cyclosporine, tacrolimus, and rapamycin) have in vitro activity against fungi, including *C. neoformans* (59,61,66,67). The antifungal activity of cyclosporine and tacrolimus is mediated by fungal homologs of calcineurin and that of rapamycin through complexes with TOR kinase (61,66). Mutations in calcineurin A and B genes have been shown to confer resistance to cyclosporine and tacrolimus and in FKBP12 gene, to tacrolimus and rapamycin in vitro (66). In addition, TOR I mutants of cryptococci have been identified that are resistant only to rapamycin (66). Despite high seroprevalence of cryptococcal antibodies in early childhood (68), cryptococcal infection is rare in transplant recipients. These data suggest that the immunosuppressive agents currently used may be conferring some degree of protection against *Cryptococcus*. Whether *C. neoformans* infections in patients receiving these immunosuppressive agents represent breakthrough infections due to resistant mutants, however, remains to be determined.

Although the susceptibility of transplant recipients to *C. neoformans* is well recognized, it is not known whether cryptococcal infection in these patients is newly acquired or a reactivation of latent infection. That cryptococcal disease may be due to a reactivation of latent infection is suggested by the following observations in the nontransplant setting: 1) autopsy studies have documented pulmonary granulomas containing *C. neoformans* in patients who had no history of *C. neoformans* infection (69); 2) molecular typing in African patients residing in Europe indicated that cryptococcosis resulted from a reactivation of latent infection (70); 3) serologic evidence of *C. neoformans* infection was documented in most children in New York City in early childhood, even though symptomatic infections were rare (68).

We previously reported that transplant recipients from the northeastern United States were more likely to have cryptococcosis than transplant recipients from other regions of the United States (8). This review shows that cryptococcal infections in patients from the Northeast developed significantly earlier after transplantation than in other patients. Although, there is incontrovertible evidence of primary acquisition of cryptococcosis in isolated case reports (71), our data suggest that *C. neoformans* may have a predilection for certain geographic areas and that most cryptococcal infections in transplant recipients may result from a reactivation of latent infection.

Epidemiologic studies of *C. neoformans* have been hampered by lack of sensitive and specific immunologic tests to evaluate the prevalence of latent infection. New immunoblotting assays (68,72), however, have unique implications not only for discerning whether cryptococcal infections result from reactivation or primary acquisition but also for identifying patients at high risk for reactivation or patients never exposed (who may therefore be vulnerable to primary infection).

The relative rarity of cryptococcal infections in pediatric organ transplant recipients has been noted (55). However, the precise reason for this is not known. If cryptococcosis represents reactivation of latent infection in a transplant setting and primary cryptococcal infection is acquired asymptomatically in childhood, it is plausible that pediatric transplant recipients may not yet have acquired the infection. *C. neoformans* infection is also strikingly rare in bone marrow transplant recipients, possibly because fluconazole prophylaxis is used widely for candidiasis or because thymic regeneration in bone marrow transplant recipients may render T cells more efficacious against cryptococci than T cells present in solid organ transplant recipients (Heitman J, pers. comm.).

Although various clinical manifestations have been described, molluscum contagiosum-like lesions are characteristic of cutaneous cryptococcosis in HIV-infected patients. In the transplant setting, cutaneous cryptococcal infection most frequently mimicked (and was clinically indistinguishable from) bacterial cellulitis. A unique propensity for the extremities to be the site of cutaneous cryptococcosis in transplant recipients was noted in this review; 94% of the patients with cutaneous *C. neoformans* infections had lesions on upper or lower extremities. Cutaneous cryptococcosis, however, represents disseminated infection and should be treated with systemic antifungal agents.

Elevated CSF pressure without evidence of obstructive hydrocephalus, believed to result from basilar meningitis and impaired reabsorption of CSF across arachnoid villi, has recently been recognized as an important complication of cryptococcal meningitis (73). HIV studies have shown that high baseline opening pressure in patients with cryptococcal meningitis correlated inversely and independently with survival. CSF opening pressure was recorded infrequently in organ transplant recipients. However, all 17 patients in whom such a measurement was conducted had intracranial pressure $\geq$140 mm of $H_2O$; the death rate in these patients was 8 (47%) of 17. These data underscore the need for assessing intracranial pressure in all patients with cryptococcal meningitis, including organ transplant recipients.

Overall, 72 (42%) of 172 of the transplant recipients with *C. neoformans* infection died. Preexistent renal failure was an independently significant predictor of death in transplant recipients with cryptococcosis. Renal failure has been proposed to increase the risk for cryptococcosis (62). Uremia decreased lymphocyte transformation and chemiluminescence by splenic cells in *C. neoformans*-infected mice (74).

This review summarizes the overall impact and highlights the key features of *C. neoformans* infection in organ transplant recipients. These include the effect of primary immunosuppressive agents on the clinical manifestations of cryptococcosis; geographic diversity in the incidence and onset of infection posttransplantion; and variables influencing outcome, specifically in the transplant setting. More importantly, however, we have identified a number of outstanding questions with implications relevant to elucidating the pathogenesis of *C. neoformans* infection. These questions involve the biologic basis of tissue tropism, reasons for the predominance of dermatotropic strains in recipients of tacrolimus, the role or virulence of immunosuppressive-agent resistant *C. neoformans* mutants in the transplant setting, and the relative rarity of cryptococcal infections in pediatric and bone marrow transplant recipients. We caution that a retrospective study may carry unknown bias. In this regard, our data may be considered hypotheses generating.

Dr. Husain is an infectious diseases fellow at the University of Pittsburgh Medical Center. His research interests include infections in immunocompromised hosts, in particular fungal infections in organ transplant recipients.

### References

1. Kanj SS, Welty-Wolf K, Madden J, Tapson V, Baz MA, Davis D, et al. Fungal infections in lung and heart-lung transplant recipients, report of 9 cases and review of the literature. Medicine 1996;75:142-56.
2. Paterson DL, Singh N. Invasive aspergillosis in transplant recipients. Medicine 1999;78:123-32.
3. Kusne S, Furukawa H, Abu-Elmagd K, Irish W, Rakela J, Fung J, et al. Infectious complications after small bowel transplantation in adults: an update. Transplant Proceed 1996;5:2761-2.
4. Benedetti E, Gruessner A, Troppmann C, Papalois BE, Sutherland DER, Dunn DL, et al. Intra-abdominal fungal infections after pancreatic transplantation: incidence, treatment, and outcome. J Am Coll Surg 1996;183:307-16.
5. Singh N. Antifungal prophylaxis in organ transplant recipients: seeking clarity amidst controversy. Clin Infect Dis 2000;31:545-53.
6. Chugh KS, Sakhuja V, Jain S, Singh V, Tarafdar A, Joshi K, et al. Fungal infections in renal allograft recipients. Transplant Proc 1992;24:1940-2.
7. Jabbour N, Reyes J, Kusne S, Martin M, Fung J. Cryptococcal meningitis after liver transplantation. Transplantation 1996;61:146-67.
8. Singh N, Gayowski T, Wagener MM, Marino IR. Clinical spectrum of invasive cryptococcosis in liver transplant recipients receiving tacrolimus. Clin Transplant 1997;11:66-70.
9. Carlson KC, Mehlmauer M, Evans S, Chandrasoma P. Cryptococcal cellulitis in renal transplant recipients. J Am Acad Dermatol 1987;17:469-72.
10. Saag MS, Powderly WG, Cloud GA, Robinson P, Grieco MH, Sharkey PK, et al. Comparison of amphotericin with fluconazole in the treatment of acute AIDS-associated cryptococcal meningitis. N Engl J Med 1992;326:83-9.
11. Zuger A, Louie E, Holzman RS, Simberkoff MS, Rahal JJ. Cryptococcal disease in patients with the acquired immunodeficiency syndrome. Diagnostic features and outcome of treatment. Ann Intern Med 1986;104:234-40.
12. Chuck SL, Sande MA. Infection with *Cryptococcus neoformans* in the acquired immunodeficiency syndrome. N Engl J Med 1989;321:794-9.
13. Shaariah W, Morad Z, Suleiman AB. Cryptococcosis in renal transplant recipients. Transplant Proc 1992;24:1898-9.
14. Biswas J, Gopal L, Sharma T, Parikh S, Madhavan HN, Badrinath SS. Recurrent cryptococcal choroiditis in a renal transplant patient. Retina 1998;18:273-6.
15. Scully RE, Mark EJ, McNeely WF, McNeely BU. Case records of the Massachusetts General Hospital. Case 7-1994. N Engl J Med 1994;330:490-6.
16. Parisi A, Sacchi P, Filice G. Treatment of cryptococcal meningitis in liver transplantation. Infection 1998;26:314-5.
17. Kaben U. Cryptoccosis of the skin. Hautarzt 1989;40:31-3.
18. Conti D, Tolkoff-Rubin NE, Baker GP Jr, Doran M, Cosimi AB, DelMonico F, et al. Successful treatment of invasive fungal infection with fluconazole in organ transplant recipients. Transplantation 1989;48:692-5.

19. O'Neil KM, Ormsby AH, Prayson RA. Cryptococcal myositis: a case report and review of the literature. Pathology 1998;30:316-7.
20. John GT, Mathew M, Snehaltha E, Anandi V, Date A, Jacob CK, et al. Cryptococcosis in renal allograft recipients. Transplantation 1994;58:855-6.
21. Sinott JT, Holt DA. Cryptococcal pyarthrosis complicating gouty arthritis. South Med J 1989;82:1555-6.
22. Leff RD, Smith EJ, Aldo-Benson MA, Arnoff GR. Cryptococcal arthritis after renal transplantation. South Med J 1981;74:1290.
23. Britt RH, Enzmann DR, Remington JS. Intracranial infection in cardiac transplant recipients. Ann Neurol 1981;9:107-19.
24. Dauber JH, Paradis IL, Dummer JS. Infectious complication in pulmonary alograft recipients. Clin Chest Med 1990;11:291-308.
25. Hall JC, Brewer JH, Crouch TT, Watson KR. Cryptoccal cellulitis with multiple sites of involvement. J Am Acad Dermatol 1987;17:329-32.
26. Jennings III HS, Bradsher RW, McGee ZA, Johnson HK, Alford RH. Acute cryptococcal cellulitis in renal transplant recipients. South Med J 1981;74:1150-3.
27. Shrader SK, Watts JC, Dancik JA, Band JD. Disseminated cryptococcosis presenting as cellulitis with necrotizing vasculitis. J Clin Microbiol 1986;24:860-2.
28. Anderson DJ, Schmidt C, Goodman J, Pomeroy C. Cryptococcal disease presenting as cellulitis. Clin Infect Dis 1992;14:666-72.
29. Gloster HM Jr, Swerlick RA, Solomon AR. Cryptococcal cellulitis in a diabetic, kidney transplant patient. J Am Acad Dermatol 1994;30:1025-6.
30. Hoston JR, Pedley TA. The neurological complications of cardiac transplantation. Brain 1976;99:673-94.
31. Kapoor A, Flenchner SM, O'Malley K, Paolone D, File TM Jr, Cutrona AF. Cryptococcal meningitis in renal transplant patients associated with environmental exposure. Transplant Infect Dis 1999;1:213-7.
32. Ooi HS, Chen BTM, Cheng HL, Khoo OT, Chan KT. Survival of a patient transplanted with a kidney infected with *Cryptococcus neoformans*. Transplantation 1971;11:428-9.
33. Beine JP, Lontie M, Vandenpitte J. Cryptococcal meningoencephalitis and 5-fluorocytosine. BMJ 1971;2:107.
34. Agut H. Puzzles concerning the pathogenicity of human herpesvirus-6 [Editorial]. N Engl J Med 1994;329:203-4.
35. Mayers DL, Martone WJ, Mandell GL. Cutaneous cryptococcosis mimicking Gram-positive cellulitis in a renal transplant patient. South Med J 1981;74:1032-3.
36. Lye WC, Chin NK, Lee YS. Disseminated cryptococcosis presenting with a pleural effusion in a kidney transplant recipient: early diagnosis by pleural biopsy and successful treatment with oral fluconazole. Nephron 1993;65:646.
37. Gallis HA, Berman RA, Cate TR, Hamilton JD, Caullie Gunnells J, Stickel DL. Fungal infection following renal transplantation. Arch Intern Med 1975;135:1163-72.
38. Mishima T, Kobayashi Y, Ohkubo M, Marumo F, Yoshimura H, Uchida H, et al. A case of renal transplant recipient complicated with cryptococcosis and amphotericin B induced acute tubular necrosis. Jpn Circ J 1977;41:1009-13.
39. Bach MC, Sahyoun A, Adler JL, Schlesinger RM, Breman J, Madras P, et al. High incidence of fungus infections in renal transplantation patients treated with antilymphocyte and conventional immunosuppression. Transplant Proc 1973;5:549-53.
40. Tipple J, Haywood H, Lee HM, Duma RJ. Cryptoccosis in renal transplant patients. Proc Clin Dial Transplant Forum 1976;6:13-9.
41. Murphy JF, McDonald FD, Dawson M, Reite A, Turcotte J, Fekety R Jr. Factors affecting the frequency of infection in renal transplant recipients. Arch Intern Med 1976;136:670-7.
42. Duston M, McHenry MC, Braun WE, Fieker DH, Gavan TL, Novick AC. Cryptococcal meningitis causing fever of unknown origin in renal transplant recipients. Report of two cases initially diagnosed by urine cultures. Transplantation 1981;32:334-6.
43. Krajewski S. Cryptococcal meningoencephalitis as a resultof long-term immunosuppression after kidney transplantation. Neuropathol Pol 1982;20:495-503.
44. Marcus JR, Hussong JW, Gonzalez C, Dumanian A. Risk factors in necrotizing fascitis: a case involving *Cryptococcus neoformans*. Ann Plast Surg 1998;40:80-3.
45. Tilney N, Kohler TR, Strom TB. Cerebralmeningitis in immunosuppressed recipients of renal allografts. Ann Surg 1982;195:104-9.
46. Watson AJ, Whelton A, Russell RP. Cure of cryptococcemia and preservation of graft function in a renal transplant recipient. Arch Intern Med 1984;144:1877-8.
47. Schroter GPJ, Temple DR, Hunsbert BS, Weill R III, Starzl TE. Cryptoccosis after renal transplantation: report of ten cases. Surgery 1976;79:268-77.
48. Plunkett JM, Turner BI, Tallent MB, Johnson K. Cryptococcal septicemia associated with urologic instrumentation in a renal allograft recipient. J Urol 1981;125:241-2.
49. Nampoory MRN, Khan ZU, Johny KV, Constandi JN, Gupta RK, Al-Muzairi I, et al. Invasive fungal infections in renal transplant recipients. J Infect 1996;33:95-101.
50. Horrevorts AM, Huysmans FTM, Koopman RJJ, Meis JFGM. Cellulitis as first clinical presentation of disseminated cryptococcosis in renal transplant recipients. Scand J Infect Dis 1994;26:623-6.
51. Hellman RN, Hinrichs J, Sicard G, Hoover R, Golden P, Hoffsten P. Cryptococcal pyelonephritis and disseminated cryptococcosis in a renal transplant recipient. Arch Intern Med 1981;141:128-30.
52. Page B, Thervet E, Legendre C, Kreis H. Cryptococcosis after renal transplantation. Transplant Proc 1999;27:1732.
53. van den Elshout FJJ, Huysmans FThM, Muytjens HL, Koene RAP. *Cryptococcus neoformans* meningitis following renal transplantation. Neth J Med 1987;31:183-90.
54. Conces DJ, Vix VA, Tarver RD. Pleural cryptococcosis. J Thorac Imag 1990;5:84-6.
55. Singh N, Rihs JD, Gayowski T, Yu VL. Cutaneous cryptococcosis mimicking bacterial cellulitis in a liver transplant recipient: case report and review in solid organ transplant recipients. J Clin Transplant 1994;8:365-8.
56. Case records of the Massachusetts General Hospital. Weekly clinicopathological exercises. N Engl J Med 1999;340:1981-8.
57. Valero G, Graybill JR. Successful treatment of cryptococcal meningitis with amhotericin B colloidal dispersion: report of four cases. Antimicrob Agent Chemother 1995;39:2588-90.
58. Granier F, Kanitakis J, Hermier C, Zhu YY, Thivolet J. Localized cutaneous cryptococcosis successfully trated with ketoconazole. J Am Acad Dermatol 1987;16:243-9.
59. Odom A, Del Poeta M, Perfect J, Heitman J. The immunosuppressant FK506 and its nonimmunosuppressive analog L-685, 818 are toxic to *Cryptococcus neoformans* by inhibition of a common target protein. Antimicrob Agent Chemother 1997;41:156-61.
60. Cardenas ME, Muir RS, Breuder T, Heitman J. Targets of immunophilin-immunosuppressant complexes are distinct highly conserved regions of calcineurin A. EMBO J 1995;14:2772-83.
61. Cruz MC, Del Poeta M, Wang P, Wenger R, Zenke G, Quesniaux VFJ, et al. Immunosuppressive and nonimmunosuppressive cyclosporine analogs are toxic to the opportunistic fungal pathogen *Cryptococcus neoformans* via cyclophilin-dependent inhibition of calcineurin. Antimicrob Agent Chemother 2000;44:143-9.
62. Perfect JR. Cryptococcosis. Infect Dis Clin North Am 1989;3:77-102.
63. Van Custem J, Fransen J, Van Gerven E, Janssen PAJ. Experimental cryptococcosis: dissemination of *Cryptococcus neoformans* and dermatropism in guinea pigs. Mykosen 1986;29:561-5.
64. Dixon DM, Polak A. *In vivo* and *in vitro* studies with atypical, rhinotropic isolate of Cryptococcus neoformans. Mycopathologia 1986;96:33-40.
65. Dromer F, Mathoulin S, Dupont B, Letenneur L, Ronin O. Individual and environmental factors associated with infection due to *Cryptococcus neoformans* serotype D. Clin Infect Dis 1996;23:91-6.

66. Cruz MC, Cavallo LM, Gorlach JM, Cox G, Perfect JR, Cardenas ME, et al. Rapamycin antifungal action is mediated via conserved complexes with FKBP12 and TOR kinase homologs in *Cryptococcus neoformans*. Mol Cell Biol 1999;19:4101-12.
67. Lorens MC, Heitman J. TOR mutations confer rapamycin resistance by preventing interaction with FKBP12-rapamycin. J Biol Chemother 2000;270:27531-7.
68. Goldman DL, Khine H, Abadi J, Lindenberg DJ, Pirofski L, Niang R, et al. Serologic evidence for *Cryptococcus neoformans* infection in early childhood. Pediatrics. In press 2001.
69. Haugen RK, Baker RD. The pulmonary lesions in cryptococcosis with special reference to subpleural nodules. Am J Clin Pathol 1954;37:1381-90.
70. Garcia-Hermoso D, Janbon G, Dromer F. Epidemiological evidence for dormant *Cryptococcus neoformans* infection. J Clin Microbiol 1999;37:3204-9.
71. Nosanchuk JD, Shoham S, Fries BC, Shapiro DS, Levitz SM, Casadevall A. Evidence of zoonotic transmission of *Cryptococcus neoformans* from a pet cockatoo to an immunocompromised patient. Ann Intern Med 2000;132:205-8.
72. Chen L-C, Goldman DL, Doering T, Pirofski L, Casadevall A. Antibody response to *Cryptococcus neoformans* proteins in rodents and humans. Infect Immun 1999;67:2218-24.
73. Graybill JR, Sobel J, Saag M, van der Horst C, Powderly W, Cloud G, et al. Diagnosis and management of increased intracranial pressure in patients with AIDS and Cryptococcal meningitis. Clin Infect Dis 2000;30:47-54.
74. Fromtling RA, Fromtling AM, Staib S, Muller S. Effect of uremia on lymphocyte transformation and chemiluminescence by spleen cells of normal and *Cryptococcus neoformans*-infected mice. Infect Immun 1981;32:1073-8.
